# Tumor necrosis factor gene polymorphisms are associated with silicosis: a systemic review and meta-analysis

**DOI:** 10.1042/BSR20181896

**Published:** 2019-02-05

**Authors:** Min Zhang, Ling-Long Peng, Xue-Lei Ji, Hai-Bing Yang, Ri-Sheng Zha, Guo-Ping Gui

**Affiliations:** 1 Central Office, Suzhou National New and Hi-tech Industrial Development Zone Center for Disease Control and Prevention, Suzhou, Jiangsu 215011, China; 2 Department of Science and Education, The Second People’s Hospital of Wuhu, Wuhu, Anhui 241000, China; 3 Department of Environmental Health, Suzhou Municipal Center for Disease Control and Prevention, Suzhou, Jiangsu 215004, China

**Keywords:** meta-analysis, silicosis, susceptibility, TNF

## Abstract

Studies investigating association between tumor necrosis factor (TNF) gene polymorphisms and silicosis susceptibility report conflicting results. The aim of this meta-analysis was to assess association between TNF gene polymorphisms and silicosis susceptibility. A systematic literature search was conducted to find relevant studies. Pooled odds ratios (ORs) with 95% confidence intervals (CIs) were used to estimate the strength of association. Finally, a total of 12 articles, involving 1990 silicosis patients and 1898 healthy controls were included in the meta-analysis. Overall, meta-analysis revealed a significant association between the TNF −308A allele and silicosis (OR = 1.348, 95%CI = 1.156–1.570, *P*<0.001). A significant association of AA+AG genotype of the TNF −308 A/G polymorphism with susceptibility to silicosis was also found (OR = 1.466, 95%CI = 1.226–1.753, *P*<0.001). After stratification by ethnicity, significant associations were detected under the genetic models (A allele and AA+AG genotype) for TNF −308A/G polymorphisms in the Asian population (*P*<0.05). Similarly, meta-analysis of the TNF −238A/G polymorphism revealed the same pattern as that shown by meta-analysis of TNF −308A/G. The meta-analysis suggests that the TNF −308A/G and −238A/G polymorphisms are associated with susceptibility to silicosis, especially in Asians.

## Introduction

Silicosis is an interstitial lung disease prevalent among miners, sand blasters, and quarry workers, manifested as a chronic inflammatory response leading to severe pulmonary fibrotic changes [1]. It is characterized by fibrotic nodules, thickening of the alveolar interstitium, and accumulation of inflammatory cells in the lung. This disease is considered a major public health problem in some developing countries such as India, South African, and China. Although the clear pathogenesis of silicosis has not been fully elucidated, evidence suggests that increased cumulative silica exposure promoted risk of silicosis [2,3]. Interestingly, it is quite pronounced that individual specific responses to dust exposure demonstrated some genes and genes variations mainly influenced the silicosis susceptibility, suggesting that genetic factors may influence susceptibility to this disease [4–6].

Silicotic inflammation and fibrosis are developed when alveolar phagocytes contact or ingest silica particles. Cytokines and fibrogenic mediators were released into the local tissues and triggered an inflammatory response, followed by fibroblast proliferation and collagenization [7]. In this regard, tumor necrosis factor (TNF)-α derived from alveolar macrophages in the lung is important in regulating these mediators in silicosis. TNF-α is an important pro-inflammatory cytokine, secreted primarily by mononuclear phagocytic cells [8]. It is involved in various physiologic and pathologic processes, such as inflammation initiation, immuno-regulation, proliferation, and apoptosis [9]. TNF-α deficient mice are resistant to developing fibrosis from silica [10] and murine lung transfected with TNF-α resulted in spontaneous alveolitis, alveolar disruption, and a progressive fibrotic reaction [11], suggesting that TNF-α may play an important role in silicosis. TNF gene is located on chromosome 6 (region p21.3), within the central major histocompatibility complex. Studies have indicated that TNF-α production is regulated at the transcriptional level [12–14], and a G-to-A mutation in the −238, −308 promoter section is accompanied by an increase in TNF-α production. Recently, studies have demonstrated that these two polymorphisms are related to the occurrence and development of silicosis [15–17]. On the contrary, some studies did not find any associations between the TNF polymorphisms (−308A/G, −238A/G) and silicosis [18–20].

Meta-analysis is a statistical method for combining the results of several studies to produce a single estimate of the major effect with enhanced precision. It is considered a powerful tool for pooling inconsistent results from different studies [21]. Li et al. performed a meta-analysis to assess association between TNF gene polymorphisms and silicosis susceptibility, but this meta-analysis included TNF −308A/G polymorphism and only nine studies [22]. More studies concerning the association between TNF polymorphisms and silicosis risk have been reported in recent years [19,23–24]. Thus, it seems necessary to perform a meta-analysis that includes the most updated data to investigate the relationship between TNF gene polymorphisms and the risk of silicosis.

## Materials and methods

### Search strategy

A systematic literature search in PubMed database, Elsevier Science Direct, the China National Knowledge Infrastructure database (CNKI), the Chinese Biomedical database (CBM), Wanfang Database, and VIP Chinese journal database was performed to identify articles. The text words were as followings: “silicosis” and “tumor necrosis factor or tumor necrosis factor gene or TNF-α or TNF alpha” combined with “gene polymorphism or polymorphisms or mutation or variant or gene.” The languages were limited to English and Chinese. The last search was updated on July 1, 2018. References in the identified studies were reviewed to find additional studies regarding the association between TNF gene polymorphisms and silicosis susceptibility.

### Inclusion and exclusion criteria

The inclusion criteria were defined as: (1) the design was a case–control; (2) the studies evaluated the association between TNF gene polymorphisms (−308A/G and −238A/G) and susceptibility to silicosis; and (3) the studies provided sufficient data to calculate the odds ratio (OR). Studies were excluded if one of the followings existed: (1) the studies contained overlapping data; (2) studies included family members who had been studied because of analysis based on linkage considerations.

### Quality assessment

The Newcastle–Ottawa scale (NOS) was adopted to assess the quality of eligible studies from three aspects: (1) selection: 0–4 points; (2) comparability: 0–2 points; and (3) outcome: 0–3 points [25]. Total scores ranged from 0 to 9, and studies with a score of more than 7 were regard as of high quality. In our meta-analysis, the scores of included studies ranged from 7 to 8, which showed that all studies included were in compliance with high quality (Table 1).

**Table 1:** Characteristics of the individual studies included in the meta-analysis

First author, year	Population (ethnicity)	Case	Control	Genotyping methods	Association		*P* (HWE)	NOS score
					*P* value (allele contrast)			
Yucesoy 2001 [1]	American (C)	325	164	PCR-RFLP	TNF −308A/G	*P*>0.05	0.003	8
					TNF −238A/G	*P*<0.05	0.127	
Crobett 2002 [30]	South African (AF)	121	120	PCR-RFLP	TNF −308A/G	*P*>0.05	0.054	8
					TNF −238A/G	*P*<0.05	0.152	
Li 2004 [18]	Chinese (A)	259	341	PCR-RFLP	TNF −308A/G	*P*>0.05	0.554	8
Wang 2005 [15]	Chinese (A)	96	116	PCR-RFLP	TNF −308A/G	*P*<0.05	0.161	7
					TNF −238A/G	*P*<0.05	0.888	
Qu 2007 [19]	Chinese (A)	184	111	PCR-RFLP	TNF −308A/G	*P*>0.05	0.105	8
Wang 2005 [16]	Chinese (A)	75	137	PCR-RFLP	TNF −308A/G	*P*<0.01	0.176	7
					TNF −238A/G	*P*<0.01	0.897	
Wu 2007 [20]	Chinese (A)	183	111	PCR-RFLP	TNF −308A/G	*P*>0.05	0.106	8
					TNF −238A/G	*P*>0.05	0.619	
Yu 2009 [29]	Chinese (A)	259	341	PCR-RFLP	TNF −238A/G	*P*>0.05	0.379	8
Rad 2012 [28]	Iranian (C)	45	45	PCR	TNF −308A/G	*P*>0.05	0.584	8
Wang 2012 [17]	Chinese (A)	68	68	PCR-RFLP	TNF −308A/G	*P*<0.05	0.945	8
					TNF −238A/G	*P*<0.05	0.951	
Kurniawidjaja 2014 [24]	Indonesian (A)	171	165	PCR-RFLP	TNF −308A/G	*P*<0.05	0.719	8
Mai 2014 [23]	Chinese (A)	64	44	PCR-RFLP	TNF −308A/G	*P*>0.05	0.450	8
					TNF −238A/G	*P*>0.05	0.877	

C: Caucasian, A: Asian, AF: African.

### Data extraction

Data were collected by two independent investigators. If these two investigators could not reach a consensus, disagreements were discussed and resolved by a third investigator. The characteristics of the selected articles are shown in Table 1, including first author, year of publication, study population, ethnicity, number of cases, controls, and findings about the polymorphisms investigated in these studies, *P* values of Hardy–Weinberg equilibrium (HWE) for controls and NOS score. The study populations comprise American, South African, Iranian, Indonesian, and Chinese. The Asian subgroup included Chinese and Indonesian populations. American and Iranian populations were classified in the Caucasian subgroup, South African in the African subgroup.

### Statistical analysis

Allele frequencies at the TNF gene polymorphisms from the individual study were determined by the counting method. The association between TNF (–308 A/G and –238 A/G) polymorphism and the risk of silicosis was estimated by calculating a pooled OR and 95%CI under an allele model (A vs. G), a recessive model (AA vs. AG+GG), a dominant model (AA+AG vs. GG), a homozygote model (AA vs. GG) and a heterozygote model (AG vs. GG).

The *χ*^2^ test-based Q statistic was used to examine the heterogeneity of between-studies [26]. The *I*^2^ statistic measures the degree of inconsistency in the studies by computing what percentage of the total variation across studies was due to heterogeneity rather than by chance. A high *I*^2^ value indicated a higher probability of the existence of heterogeneity (*I*^2^ = 0–25%, no heterogeneity; *I*^2^ = 25–50%, moderate heterogeneity; *I*^2^ = 50–75%, large heterogeneity; and *I*^2^ = 75–100%, extreme heterogeneity). If the *P* value of the heterogeneity Q statistic was less than 0.10, the random effects model was selected. Otherwise, a fixed effects model was adopted. Publication bias was estimated using Egger’s linear regression test and a funnel plot. If the *P* value was less than 0.05, statistically significant publication bias might exist [27].

All the statistical analyses for the meta-analysis were performed with STATA statistical software (version 11.0 STATA Corp, College Station, TX). A two-sided *P* value <0.05 was regarded as statistically significant.

## Results

### Literature search and study characteristics

**Figure 1:**
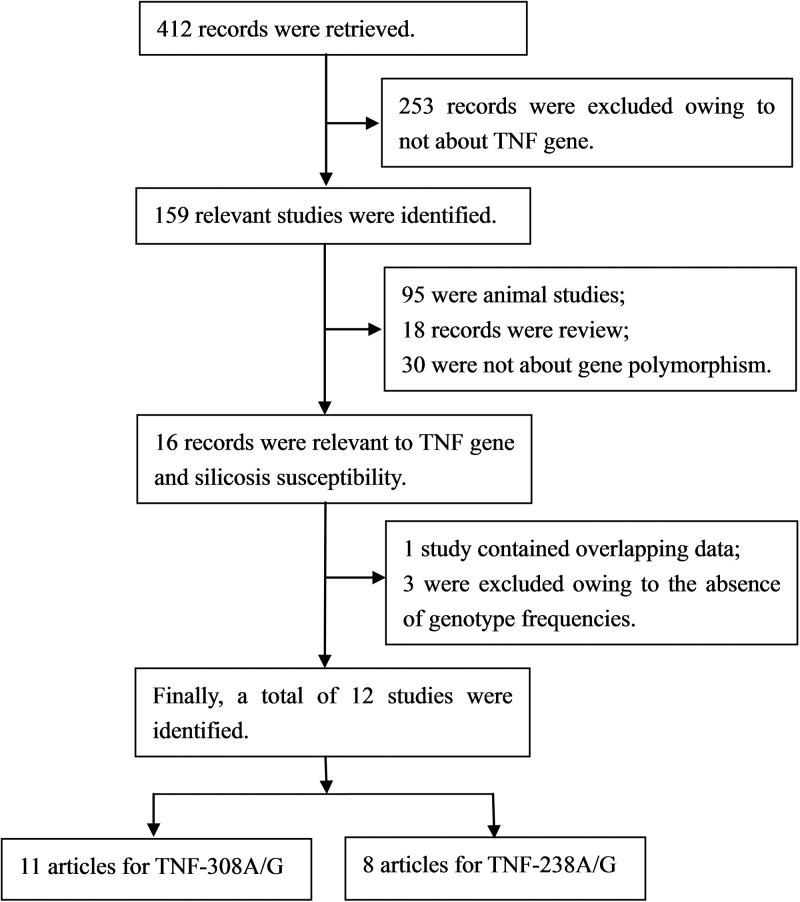
Flow diagram of the study selection process

The process for selecting the studies is shown in Figure 1. The initial search of databases identified 412 potentially relevant studies of which 412 articles, 253 records were excluded owing to not about TNF gene, 95 were animal studies, 18 records were review, 30 were not about gene polymorphisms, 1 study contained overlapping data, and 3 were excluded owing to the absences of genotype frequencies. Finally, 12 articles met the inclusion criteria and were finally included in our meta-analysis [1,15–20,23,24,28–30] including 1990 patients with silicosis and 1898 healthy controls. There were 11 studies on TNF −308A/G, 8 studies involved Asian populations [15–20,23,24], 2 studies involved Caucasian populations [1,28], and 1 study involved African populations [30]. There were 8 studies on TNF −238A/G, 6 studies involved Asian populations [15–17,20,23,29], 1 study involved Caucasian populations [1], and 1 study involved African populations [30]. The main characteristics of each study included in this meta-analysis are shown in Table 1.

### Evaluation of heterogeneity and publication bias

Heterogeneity of the included studies regarding each polymorphism is presented in Table 2. Between-study heterogeneity was found during some meta-analyses of TNF −238A/G polymorphism in the overall population and the Asian group. So these meta-analyses were performed in a random effects model, and the other meta-analyses were done in a fixed effects model.

**Table 2:** Meta-analysis of TNF gene polymorphisms and silicosis

Polymorphisms	Populations	Number of studies	Sample size		Test of association				Test of heterogeneity			Egger’s test (*P*)
			Case	Control	OR (95%CI)	*Z*	*P*	Model	*χ*^2^	*P*	*I^2^*	
TNF −308A/G	Overall	11	1591	1422	1.348 (1.156–1.570)	3.82	0.000	F	11.59	0.314	13.7%	0.016
A vs. G		NA	NA	NA	1.158 (1.012–1.325)^a^	NA	NA	NA	NA	NA	NA	NA
	Asian	8	1100	1093	1.581 (1.288–1.941)	4.83	0.000	F	5.33	0.619	0.0%	0.079
	Caucasian	2	370	209	1.183 (0.897–1.559)	1.19	0.234	F	0.10	0.757	0.0%	NA
	African	1	121	120	0.923 (0.605–1.407)	0.37	0.709	NA	NA	NA	NA	NA
AA vs. AG+GG	Overall	11	1591	1422	1.194 (0.752–1.898)	0.75	0.452	F	5.93	0.821	0.0%	0.009
		NA	NA	NA	0.811 (0.536–1.228)^a^	NA	NA	NA	NA	NA	NA	NA
	Asian	8	1100	1093	1.689 (0.894–3.226)	1.62	0.106	F	2.32	0.940	0.0%	0.148
	Caucasian	2	370	209	1.024 (0.382–2.744)	0.05	0.962	F	0.95	0.331	0.0%	NA
	African	1	121	120	0.608 (0.228–1.627)	0.99	0.322	NA	NA	NA	NA	NA
AA+AG vs. GG	Overall	11	1591	1422	1.466 (1.226–1.753)	4.19	0.000	F	8.31	0.598	0.0%	0.086
	Asian	8	1100	1093	1.640 (1.307–2.058)	4.27	0.000	F	5.19	0.637	0.0%	0.118
	Caucasian	2	370	209	1.318 (0.925–1.878)	1.53	0.127	F	0.00	0.986	0.0%	NA
	African	1	121	120	1.021 (0.609–1.712)	0.08	0.936	NA	NA	NA	NA	NA
AA vs. GG	Overall	11	1591	1422	1.321 (0.826–2.112)	1.16	0.245	F	6.15	0.802	0.0%	0.010
		NA	NA	NA	1.209 (0.730–2.002)^a^	NA	NA	NA	NA	NA	NA	NA
	Asian	8	1100	1093	1.899 (0.995–3.624)	1.95	0.052	F	2.42	0.933	0.0%	0.196
	Caucasian	2	370	209	1.146 (0.417–3.149)	0.26	0.792	F	0.78	0.376	0.0%	NA
	African	1	121	120	0.636 (0.234–1.733)	0.88	0.376	NA	NA	NA	NA	NA
AG vs. GG	Overall	11	1591	1422	1.472 (1.222–1.772)	4.08	0.000	F	6.53	0.769	0.0%	0.205
	Asian	8	1100	1093	1.611 (1.272–2.040)	3.96	0.000	F	4.79	0.686	0.0%	0.235
	Caucasian	2	370	209	1.325 (0.925–1.900)	1.53	0.125	F	0.05	0.830	0.0%	NA
	African	1	121	120	1.139 (0.655–1.980)	0.46	0.645	NA	NA	NA	NA	NA
TNF −238A/G	Overall	8	1191	1101	2.092 (1.232–3.550)	2.73	0.006	R	21.76	0.003	67.8%	0.061
A vs. G	Asian	6	745	817	3.044 (1.234–7.510)	2.42	0.016	R	20.70	0.001	75.8%	0.052
	Caucasian	1	325	164	1.353 (0.994–1.840)	1.92	0.055	NA	NA	NA	NA	NA
	African	1	121	120	1.339 (0.554–3.239)	0.65	0.517	NA	NA	NA	NA	NA
AA vs. AG+GG	Overall	8	1191	1101	1.374 (0.562–3.362)	0.70	0.486	F	1.90	0.755	0.0%	0.018
		NA	NA	NA	1.027 (0.440–2.402) ^a^	0.57	NA	NA	NA	NA	NA	NA
	Asian	6	745	817	3.934 (0.614–25.193)	1.45	0.148	F	0.70	0.966	0.0%	0.567
	Caucasian	1	325	164	0.886 (0.256–3.074)	0.19	0.849	NA	NA	NA	NA	NA
	African	1	121	120	0.992 (0.137–7.156)	0.01	0.993	NA	NA	NA	NA	NA
AA+AG vs. GG	Overall	8	1191	1101	2.230 (1.297–3.836)	2.90	0.004	R	19.22	0.006	64.9%	0.091
	Asian	6	745	817	3.063 (1.240–7.567)	2.43	0.015	R	19.88	0.001	74.8%	0.051
	Caucasian	1	325	164	1.631 (1.112–2.392)	2.50	0.012	NA	NA	NA	NA	NA
	African	1	121	120	1.454 (0.535–3.956)	0.73	0.463	NA	NA	NA	NA	NA
AA vs. GG	Overall	8	1191	1101	1.635 (0.666–4.016)	1.07	0.283	F	1.80	0.772	0.0%	0.044
		NA	NA	NA	1.220 (0.549–2.708)^a^	NA	NA	NA	NA	NA	NA	NA
	Asian	6	745	817	4.591 (0.716–29.452)	1.61	0.108	F	0.07	0.965	0.0%	0.682
	Caucasian	1	325	164	1.145 (0.325–4.027)	0.21	0.833	NA	NA	NA	NA	NA
	African	1	121	120	1.018 (0.141–7.354)	0.02	0.986	NA	NA	NA	NA	NA
AG vs. GG	Overall	8	1191	1101	2.205 (1.299–3.743)	2.93	0.003	R	18.18	0.011	61.5%	0.093
	Asian	6	745	817	2.919 (1.222–6.976)	2.41	0.016	R	18.29	0.003	72.7%	0.052
	Caucasian	1	325	164	1.659 (1.125–2.446)	2.56	0.011	NA	NA	NA	NA	NA
	African	1	121	120	1.629 (0.517–5.132)	0.83	0.405	NA	NA	NA	NA	NA

F: fixed effects model, R: random effects model, NA: not available.

a Adjusted using the “trim and fill” method.

Evidence of publication bias was observed for the meta-analysis of the TNF −308A/G (allele contrast, recessive model, and homozygote model) and −238A/G (recessive model and homozygote model) in all study subjects. Thus, the “trim and fill” method was used to adjust for publication bias. The adjusted results using the “trim and fill” technique remained unchanged, suggesting that these results might not be affected by publication bias (Table 2).

### Meta-analysis of tumor necrosis factor gene polymorphisms in silicosis

A summary of the meta-analysis of the relationship between TNF gene polymorphisms and silicosis is listed in Table 2.

### Tumor necrosis factor −308A/G polymorphism and silicosis

**Figure 2:**
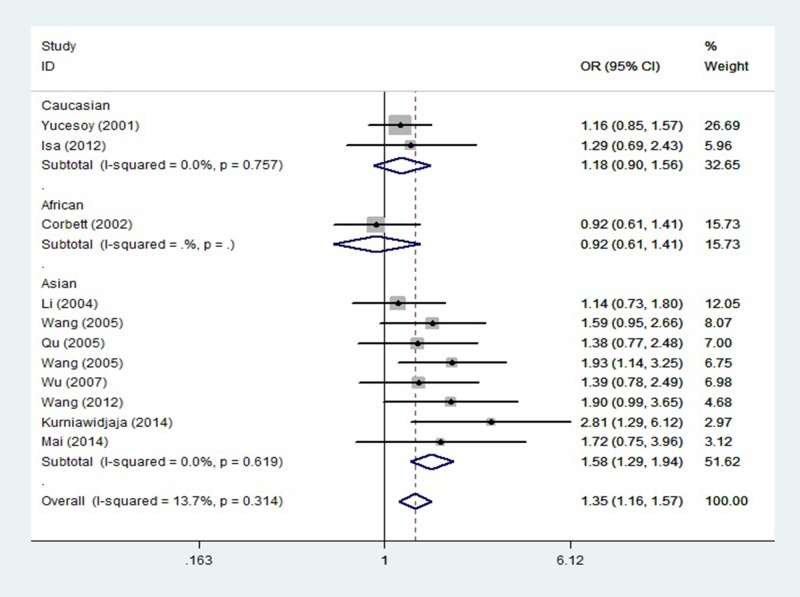
Odds ratios and 95% confidence intervals for individual studies and pooled data for the association between the A vs. G allele of TNF −308A/G polymorphism and silicosis

Eleven studies determined the relationship between the −308A/G polymorphism and silicosis. The total sample size for patients with silicosis and healthy controls was 1591 and 1422, respectively. Meta-analysis revealed a significant association between −308A allele and silicosis in the overall population (OR = 1.348, 95%CI = 1.156–1.570, *P* = 0.000, Figure 2). Analysis using dominant and heterozygote models also showed a significant association between −308A/G and silicosis risk in all study subjects (OR = 1.466, 95%CI = 1.226–1.753, *P* = 0.000 and OR = 1.472, 95%CI = 1.222–1.772, *P* = 0.000, respectively). Stratification by ethnicity indicated that −308A/G polymorphism was significantly associated with silicosis risk in the Asian population (A vs. G: OR = 1.581, 95%CI = 1.288–1.941, *P* = 0.000; AA+AG vs. GG: OR = 1.640, 95%CI = 1.307–2.058, *P* = 0.000; AG vs. GG: OR = 1.611, 95%CI = 1.272–2.040, *P* = 0.000).

### Tumor necrosis factor −238A/G polymorphism and silicosis

**Figure 3:**
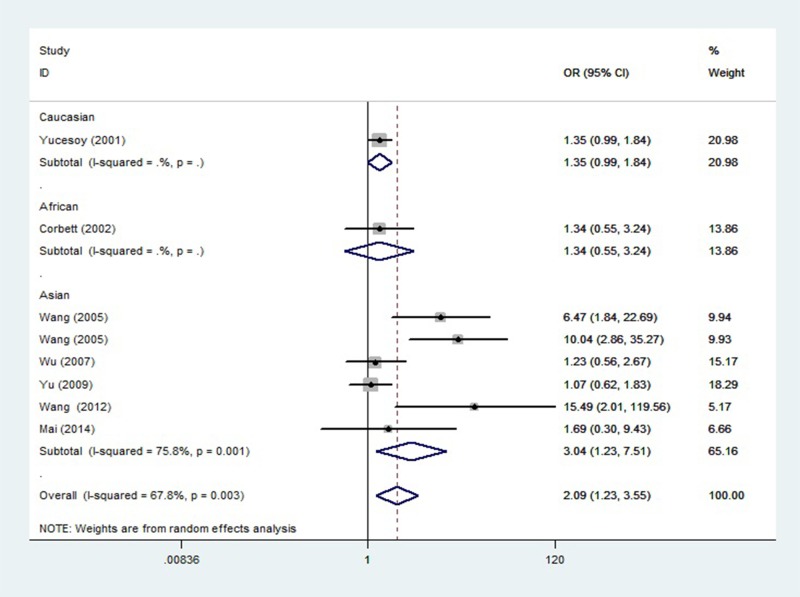
Odds ratios and 95% confidence intervals for individual studies and pooled data for the association between the A vs. G allele of TNF −238A/G polymorphism and silicosis

Eight case–control studies including 1191 cases and 1101 controls identified an association between the TNF −238A/G polymorphism and silicosis risk. The pooled OR (95%CI, *P* value) in the A vs. G allele was 2.092 (1.232–3.550, *P* = 0.006). Meta-analysis found a significant association between the AA+AG genotype and silicosis in all study subjects (OR = 2.230, 95%CI = 1.297–3.836, *P* = 0.004). Meta-analysis of heterozygote model also revealed a significant association between −238A/G and silicosis risk in all study subjects (OR = 2.205, 95%CI = 1.299–3.743, *P* = 0.003). Ethnicity-specific analysis indicated an association between −238A allele and silicosis in the Asian group (OR = 3.044, 95%CI = 1.234–7.510, *P* = 0.016). In addition, significant association were identified in the dominant and heterozygote models for the Asian silicosis population (OR = 3.063, 95%CI = 1.240–7.567, *P* = 0.015; OR = 2.919, 95%CI = 1.222–6.976, *P* = 0.016). The forest plot is shown in Figure 3.

## Discussion

Silicosis is pneumoconiosis of lung fibrosis caused by inhalation of silica particles usually at low levels but for long periods. It is a common occupational disease among the workers who are exposed to silica particles. Disease development is related to both environmental and individual factors. As is known, there is individual susceptibility to most of the human diseases even with the same environmental exposure. Host factors, including gene polymorphisms involved in the diseases, might have interpreted this difference partly. Therefore, genetic susceptibility to the diseases has been a research focus in the scientific community. It has been recognized that exposure to dust is a start-up factor, but only part of those exposed to dust get silicosis, suggesting that individual factors performed significantly in silicosis [31].

TNF-α is one of the most relevant cytokines to the biological events in silicosis such as inflammation and silica-induced pulmonary fibrosis, regulating cell proliferation, differentiation, and apoptosis [19,32,33]. Findings from animal models showed that TNF-α associated with silica-induced lung damage [34,35]. TNF pro-inflammatory cytokine overexpression and involvement in silica-induced sponge collagen biosynthesis was demonstrated in quartz-treated explants as compared with controls by means of specific TNF inhibitors affecting the fibrogenic gene response [36]. Zhang et al. have found that anti-TNF may improve silica-induced pulmonary inflammation by decreasing the TNF-α, inhibiting NF-κB signaling as well as oxidant status, which suggest that anti-TNF has potential role in the treatment of silica-induced lung damage [37]. On the other hand, several studies discussed association of TNF gene polymorphisms in patients with silicosis, but results were inconsistent. In a study with Chinese workers exposed to silica particles, there was significant correlation between polymorphisms of TNF −308A/G and −238A/G polymorphisms and risk of silicosis [17]. Similarly, in the Han population of Southwest China, TNF gene polymorphisms (−308A/G and −238A/G) might be related to occurrence of silicosis and the degree of severe pulmonary fibrosis in silicosis [15]. By contrast, polymorphisms of TNF −308A/G were not associated with silicosis in an Iranian population [28]. Therefore, to better comprehend the relationship between these two polymorphisms and silicosis, a pooled analysis with a large sample size, and heterogeneity explored is needed.

The present meta-analysis found a significant association between TNF −308A polymorphism and silicosis in the overall population, and a significant association of AA+AG genotype of TNF −308A/G polymorphism with susceptibility to silicosis was noted. Similarly, there was significant association between TNF −238A allele and all silicosis cases, and AA+AG genotype of TNF −238A/G polymorphism indicated a significant association with silicosis. However, recessive model (AA vs. AG+GG) of both TNF −308 A/G and −238A/G was not significantly related to silicosis in all populations. These findings show that TNF −308A/G and −238A/G polymorphisms might help to explain the individual differences in the susceptibility to silicosis. Considering the effect of genetic background on the results, subgroup analyses by ethnicity were performed for these polymorphisms. The present meta-analysis showed that TNF −308A/G polymorphism was related to silicosis in Asians. Dominant model of TNF −238A/G polymorphisms (AA+AG vs. GG) showed significantly increased silicosis risk for Caucasian population, but it might not be reliable because only one published article in Caucasian population was included. In addition, single nucleotide polymorphisms (SNPs) have geographical and ethnic differences [38]. Therefore, even the same polymorphisms in genes may lead to different effects on different groups of silicosis susceptibility. Consequently, the result should be interpreted with caution, and additional studies with further large-scale case–control ones, especially in Caucasian and African population are needed to validate the result.

The findings of the present study seem to contradict with some individual studies, by which those studies did not find significant correlation between TNF (−308A/G and −238A/G) polymorphisms and risk of silicosis. Reasons may be that, first, even though results of some individual studies explored correlation between TNF gene polymorphisms and risk of silicosis are not significant, the ORs (95%CIs) of the individual studies [18–20,23,27] draw near significant values as shown in Figures 2 and 3. If those individual studies increased the sample size, significant association might be revealed. Second, meta-analysis is a means of increasing the effective sample size under investigation through pooling data from individual association studies, and can overcome the limitations of individual studies, resolve inconsistencies, and reduce the likelihood that random errors are responsible for false-positive or false-negative associations, therefore, meta-analysis can enhance the statistical power of the analysis for estimating genetic effects.

Compared with the previous meta-analysis [22], the present study included 11 studies on association of −308A/G polymorphism and silicosis, and 8 studies on association of −238A/G polymorphism and silicosis, which is larger than the data from the previous meta-analysis. Furthermore, this is the first study to confirm the association between the TNF −238A/G polymorphism and silicosis susceptibility. Besides, subgroup analyses by ethnicity were performed to discuss ethnic effect of both gene polymorphisms on risk of silicosis. Thus, our meta-analysis might enhance the statistical power and draw a more reliable conclusion.

Several limitations of the present study should be considered. First, the present study could not analyze the potential gene–environment interactions and gene susceptibility haplotypes owing to lack of relevant data. Second, our literature search was dependent on English and Chinese, language bias might be considered. Third, meta-analysis remains a retrospective research, which is subject to the methodological deficiencies of the included studies.

In summary, this updated meta-analysis suggests that TNF −308A/G and −238A/G polymorphisms are associated with susceptibility to silicosis. However, large sample size studies including more ethnic groups should be considered in future association studies to confirm the results of our meta-analysis.

## Abbreviations

CI: confidence interval
HWE: Hardy–Weinberg equilibrium
NOS: Newcastle–Ottawa scale
OR: odds ratio
TNF: tumor necrosis factor
